# Tofacitinib Promotes Functional Recovery after Spinal Cord Injury by Regulating Microglial Polarization via JAK/STAT Signaling Pathway

**DOI:** 10.7150/ijbs.84564

**Published:** 2023-09-11

**Authors:** Hongdao Ma, Chenfeng Wang, Lin Han, Fanqi Kong, Zhixiao Liu, Bangke Zhang, Wenxiang Chu, Haibin Wang, Liang Wang, Qisheng Li, Weilin Peng, Haisong Yang, Chaofeng Han, Xuhua Lu

**Affiliations:** 1 Department of Orthopaedics, Shanghai Changzheng Hospital, Shanghai 200003, China.; 2 Department of Orthopaedics, Third Affiliated Hospital of Naval Medical University, Shanghai, 200433, China.; 3 Department of Histology and Embryology, Naval Medical University, Shanghai 200433, China.

**Keywords:** spinal cord injury, tofacitinib, microglia, neuron, JAK/STAT pathway

## Abstract

**Background:** The JAK/STAT signaling pathway is the main inflammatory signal transduction pathway, whether JAK/STAT contributes the pathology of SCI and targeting the pathway will alleviate SCI needs to be addressed. Here, we explored the therapeutic effect of pan-JAK inhibitor tofacitinib (TOF) on secondary injury after SCI and explained the underlying mechanisms.

**Methods:** SCI model in rat was established to evaluate the therapeutic effects of TOF treatment *in vivo*. Histological and behavioral analyses were performed at different time points after SCI. *In vitro*, the effects of TOF on pro-inflammatory activation of primary microglia and BV2 cells were analyzed by western blot analysis, fluorescent staining, qPCR and flow cytometry. The neuroprotection of TOF was detected using a co-culture system with primary neurons and microglia.

**Results:** TOF can effectively improve motor dysfunction caused by spinal cord injury in rats. TOF administration in the early stage of inflammation can effectively inhibit neuronal apoptosis and scar tissue formation, and promote the repair of axons and nerve fibers. Further studies have demonstrated that TOF suppresses inflammation caused by spinal cord injury by inhibiting the activation of microglia to pro-inflammatory phenotype *in vivo* and *in vitro*. Additionally, an interesting phenomenon is revealed in our results that TOF exhibits superior neuronal protection during inflammation *in vitro*.

**Conclusions:** Our study showed that TOF could regulate microglial activation via JAK / STAT pathway and promote the recovery of motor function after SCI, which is of great significance for the immunotherapy of SCI.

## Introduction

Spinal cord injury (SCI) refers to the temporary or permanent injury of the spinal cord, resulting in changes in the spinal structure and function. The pathological mechanisms of SCI include primary spinal cord injury and secondary spinal cord injury 1. Primary SCI occurs when an external mechanical violence causes spinal fracture or displacement, forming compression on the spinal cord. This is often followed by a series of delayed injuries known as secondary SCI, which involves complex physiological and biochemical cascade reactions, including microenvironmental changes such as hematoma, edema, inflammatory reaction, free radical accumulation, and local ischemia; destruction of the blood-spinal cord barrier; neuronal apoptosis and demyelination. As a result, the injury expands in scope and severity, forming glial scar and cavity, resulting in permanent neurological dysfunction 1, 2.

Immune response is an important pathological mechanism in secondary SCI. After SCI, a series of immune responses are immediately initiated and a variety of inflammatory stimuli are activated, leading to massive proliferation of inflammatory cells and secretion of inflammatory factors, in which the polarization of macrophages / microglia plays an important role 3-5. Studies have shown that pro-inflammatory microglia secrete oxidative metabolites and inflammatory cytokines such as interleukin (IL)-1β, IL-6, tumor necrosis factor (TNF)-α, which generate cytotoxic effects on neurons and glia, and induce infiltration of other inflammatory cells and astrocytes. These inflammatory responses promote ongoing apoptosis of neurons, resulting in the formation of spinal cord cavity and glial scar 2, 3. The anti-inflammatory microglia, on the other hand, secrete neurotrophic factors that regulate immune responses and are generally thought to be beneficial 4, 5. The proportion of pro- and anti-inflammatory macrophage/microglia at different stages after SCI determines the outcome of SCI 6, and treatment of SCI by regulating the polarization type of microglia has been effectively verified in animal experiments 7-9.

The Janus kinase (JAK)/ signal transduction and transcriptional activator (STAT) signal axis is a central pathway that mediates cellular response to inflammation and promotes carcinogenesis. It is involved in many important biological processes including cell proliferation, differentiation, apoptosis, and immune regulation, and plays an important role in rheumatic diseases, tumors and cancers, Parkinson's disease, multiple sclerosis, and inflammatory bowel disease 10. In recent years, researchers have found that JAK/STAT signaling pathway can be activated after SCI 11, which can regulate astrocyte proliferation 12 and involved in neuroinflammation associated with polarization of microglia 13. In addition, growing numbers of neurological studies have demonstrated that drug interventions against inflammation protect neurons by inhibiting JAK/STAT pathway 14-16.

Tofacitinib (TOF), a selective inhibitor of Janus kinase (JAK) mainly targeting JAK1 and JAK3 17, has been approved as the first-line biological treatment for autoimmune diseases 18. TOF affects inflammation-associated disease progression by regulating cytokines such as IL-6, TNF-α and IFNs 19, and has been widely used for many immune disorders. Furthermore, recent pharmacological studies have demonstrated that TOF has the capability of passing through the blood-brain barrier 17, 20, and is also able to ameliorate inflammation in autoimmune encephalomyelitis 21. However, the regulation of microglia polarization and the protective function of TOF following SCI are currently unclear.

Our aim in this study was to determine whether the administration of TOF could suppress inflammatory responses and consequently improve functional recovery following SCI using a rat model and investigate the mechanisms* in vitro*. It was found that TOF effectively decreased the early inflammation levels after SCI, which in turn inhibited neuronal apoptosis and glial scar formation and promoted the recovery of lower limb motor function.* In vitro* experiments further demonstrated that this protective effect on neurons was associated with the role of TOF in regulating microglia activation through JAK / STAT pathway and reducing local inflammatory factors.

## Materials and methods

### Animals

SD male rats aged 8-10 weeks and weighing 180-200g were ordered from the Animal Experiments Center of Naval Medical University (Shanghai, China) and were housed in a specific pathogen-free condition under a standarded lightdark cycle with food and water ad libitum. All animal experiments were conducted with approval from the Animal Welfare and Research Ethics Committee of Shanghai Changzheng Hospital (Shanghai, China), in strict accordance with the guidelines of the Chinese National Institutes of Health.

### SCI model establishment and TOF treatment *in vivo*

All the rats were divided randomly into the following four groups: sham group; control group (vehicle group); low-dose TOF treatment group (TOF-5 group) and high-dose TOF treatment group (TOF-10 group). The rats in Sham group only underwent laminectomy, and the rats in the other groups were SCI modeled according to the modified Allen method under sterile conditions. Briefly, after deep anesthesia with 1% pentobarbital sodium, the spinal cord of the rat was exposed at T9-10 level by laminectomy. Then, a spinal cord impactor (RWD, CA, USA) was used to produce an injury by dropping an 8g rod from a height of 6 cm onto the dorsal surface of the spinal cord. Then the incision sites were closed in layers and topical antibiotic was applied to the incision. After surgery, the rats in treatment groups received a 10 mg/kg or 5mg/kg dose of TOF bid via the intraperitoneal cavity for 28 days, and rats in Sham and Vehicle (Veh) groups were injected with normal saline in the same way. All rats received post-surgical analgesia, and SCI rats received manual bladder voiding twice a day until the bladder reflex was recovered.

### Motor function evaluation

To evaluate Hindlimb motor function, the Basso Beattie Bresnahan (BBB) scale and the Louisville Swim Score (LSS) were used as previously described 22. Before the test, rats were placed in the open field and allowed to freely move for 5 min. Then, two independent and well-trained experimenters performed blinded assessments of their hindlimb performance using the BBB scale, which ranges from 0 (complete paralysis) to 21 (normal locomotion) according to the joint movement and crawling coordination. The evaluation for rats in each group (n=8) was performed pre-operatively, and at day 1, 3, 5, 7, 14, 21 and 28 following injuries.

The swimming test was performed to evaluate forelimb dependency, hindlimb coordination, body angel and trunk stability using the 0-15 LSS scoring system. Rats were adapted to the environment with water before the test, and trained to swim from one end to the other in a water-filled glass tank. Each rat was tested twice in a double-blind manner pre-operatively and weekly post-injury until one month.

### Tissue collection

The rats were sacrificed by an overdose of pentobarbital sodium (80 mg/kg, intraperitoneally) at specific time points post injury, and spinal cord tissues from the lesion epicenter were collected. For Western blot and ELISA assay, spinal tissue samples were homogenized in RIPA lysis buffer (P00103J Beyotime, Shanghai, China), with 1% protease inhibitors. The lysates were centrifuged at 12000g at 4℃ for 15 min and the supernatants containing protein were collected. Then, the total protein concentration was determined using a BCA kit (P0012, Beyotime).

For histologic and immunofluorescence staining, tissue sections were prepared firstly. Rats were perfused transcardially with 0.9% saline solution followed by 4% paraformaldehyde (PFA) under deep anesthesia. The injured spinal cord about 5mm long was dissected and fixed in 4% paraformaldehyde overnight. After dehydration through a sucrose gradient, the tissue was paraffin-embedded and cut into 5 μm-thick longitudinal or transverse sections for the subsequent experiments.

### Histologic staining

The paraffin sections were deparaffinized in xylene, rehydrated through an alcohol gradient, washed several times with distilled water, and then stained as follows. Briefly, for HE staining, the sections were first stained with hematoxylin for 5 min, incubated in 1% acid alcohol and blued with ammonia water, and then stained with 0.5% eosin for 1 min. For Luxol fast blue staining, the sections were incubated in LFB solution overnight at room temperature, followed by differentiation in 0.05% lithium carbonated solution and washing with 70% ethanol. These steps were repeated until the grey matter and white matter could be identified clearly. After staining, all sections were dehydrated with graded ethanol, hyalinized with xylene and sealed with neutral gum. Finally, the structures in the spinal cord were observed under a light microscope (Olympus, Tokyo, Japan).

### Immunofluorescence staining

For cell immunofluorescence staining, cells were seeded on slides and cultured in 12-well plates. After pre-treatment with TOF or the vehicle and stimulation with LPS, the slides were collected and immersed in 4% paraformaldehyde for 30 min, followed by permeabilizing with 0.5% Triton X-100. For tissue immunofluorescence staining, after the sections were deparaffinized and rehydrated, antigen retrieval was carried out with EDTA antigen repair buffer (pH 8.0). The cell slides or tissue sections treated as described above were blocked with 10% bovine serum albumin (BSA) and then incubated at 4 °C overnight with the following primary antibodies: anti-MAP2 (GB11128-2, Servicebio, Wuhan, China), anti-GAP43 (GB11095, Servicebio), anti-GFAP (GB12096, Servicebio), anti-iNOS (13120S, Cell Signaling Technology, Danvers, MA, USA), anti-Arg1 (93668S Cell Signaling Technology), anti-Iba1 (GB12105, Servicebio), anti-NeuN (GB11138, Servicebio), and anti-Cleaved- Caspase3 (9664S, Cell Signaling Technology). On the following day, the sections or slides were incubated with secondary antibodies including cy3-conjugated goat anti-mouse (GB21301, Servicebio) and Alexa Flour 488-conjugated goat anti-rabbit (GB25303, Servicebio), followed by nuclei counterstaining with 4',6-diamidino-2-phenylindole (DAPI). Finally, the immunofluorescence images were obtained by a scanning instrument (Pannoramic DESK P-MIDI P250, 3D HISTECH, Hungary). Quantification of the fluorescence was performed in three different sample regions of interest (ROI) by measuring the intensity of fluorescent signal and the percentage.

### Isolation of primary microglia and primary neurons

The primary microglia and primary neurons were extracted from the brain of newborn mice (1-3day old). In brief, after the blood vessels and meninges were removed, the brain tissues were cut into pieces and digested with 0.25% trypsin-EDTA for 15 min. Then the digested tissues were centrifuged at 1000 rpm for 6 min, and subsequently isolated microglia or neurons with different medium. For primary microglia, the mixture of cells was inoculated into poly-D-lysine precoated T25 flasks and cultured in DMEM/F12 with fetal bovine serum (FBS), penicillin, and streptomycin. After 14 days of culture *in vitro*, the flasks were shake at 200 rpm at 37 ℃ for 6h to remove the mature microglia. The microglia supernatants were collected for further experiments. For primary neurons, the cells were seeded on the poly-D-lysine pretreated plates, and incubated in neurobasal medium containing 2% B27, penicillin, and streptomycin. Neurons were collected after 7 days *in vitro* culture.

### Cell culture and treatment

Mouse microglia cell line (BV2 cells) and mouse neuron cell line (HT22 cells) were obtained from the American Type Culture Collection. All cell lines were cultured in Dulbecco's modified Eagle's medium supplemented with 10% FBS, 100 units/ ml penicillin, and 100 μg/ml streptomycin at 37 °C with 5% CO2. The DMEM medium was replaced daily and passaged when cells grew to 80% confluence. According to the CCK8 result, Primary microglia and BV2 cells were pre-treated with different doses of TOF (HY-40354A, MedChemExpress, Monmouth Junction, NJ, USA) or vehicle (dimethyl sulfoxide, DMSO, D2650, Sigma-Aldrich St Louis, MO, USA) for 4h, then the medium was changed and incubated cells with lipopolysaccharide (LPS, 100 ng/ml, L2630, Sigma-Aldrich) for 24h to induce inflammatory differentiation.

### Co-culture experiments

Prior to co-culture with primary neurons or HT22 cells, BV2 cells were seeded on transwell inserts (Corning, 0.4 μm pores, Kennebunk, ME, USA) and cultured for 1 day, followed by inducing inflammatory differentiation with LPS for 24 h. Meanwhile, neurons were seeded on the other well and pre-treated with TOF. Then, the inserts with treated BV2 cells were transferred into the neuron-containing wells. After 24 h co-culture, neurons were harvested for apoptosis and morphology assessment by flow cytometry, Western blot and immunofluorescence.

### Cytotoxicity and cell viability assay

To assess the cytotoxicity of TOF and cell viability, CCK-8 assay was performed based on the manufacturer's instruction. First, cells were seeded into a 96-well plate and cultured for 24 h, and the medium was replaced with fresh medium containing different concentrations of TOF. After 24 or 48-h incubation, 10 μL CCK-8 solution (HY-K0301, MedChemExpress) was added into each well and reacted for another 4 h. The absorbance at 450 nm was measured with a microplate spectrophotometer reader (Tecan, infinite M200 PRO, Austria).

### Flow cytometry

To analyze the apoptosis of neurons under the co-culture conditions, cells were evaluated using Annexin-V-FITC / PI apoptosis detection kit (640914, Biolegnd, San Diego, CA, USA) according to the protocol. Briefly, after two washes with ice-cold phosphate buffered saline (PBS), the adherent neurons or all neurons (including supernatant) were re-suspended in the buffer with 5 μL Annexin-V-FITC and 5 μL propidium iodide (PI), and then incubated in darkness at 37°C for 20 min. To detect microglia polarization, the treated primary microglia were collected and incubated with iNOS-APC (17-5920-80, eBioscience, San Diego, CA, USA) and F4/80-FITC (11-4801-81, eBioscience) for 30 min at 4°C. After all processing, the data were measured by flow cytometry (Beckman, Cytoflex, USA) and analyzed by FlowJo software (Version 10, Treestar, Ashland, OR, USA).

### Western blot analysis

According to the molecular weight of the target protein, cells or tissue samples containing the same amount of protein were loaded onto appropriate concentration SDS-PAGE for electrophoretic separation, and subsequently transferred to the PVDF membrane (Millipore, Billerica, MA, USA), which was then blocked in the blocking buffer (Epizyme, Shanghai, China) for 1 h at room temperature, followed by incubation overnight at 4 °C with the following primary antibodies: anti-β-actin (AC038, Abclonal, Wuhan, China), anti-STAT1 (AF6300, Affinity Biosciences, Changzhou, China), anti-phospho-STAT1 (AF3300, Affinity), anti-STAT3 (AF6294, Affinity), anti-phospho-STAT3 (AF3293, Affinity), anti-Bcl2 (A0208, Abclonal), anti-Cleaved-Caspase3 (9664S, Cell Signaling Technology), anti-GFAP (GB12096, Servicebio), anti-MAP2 (A0453, Abclonal), anti-Bax (A19654, Abclonal), anti-iNOS (13120S, Cell Signaling Technology), and anti-Arg1 (93668S, Cell Signaling Technology). On the following day, the membrane was incubated with relevant HRP-conjugated secondary antibody (S0001, S0002, Affinity) for 2 h, and visualized using ECL reagent (SD6032, Simuwu, Shanghai, China). The protein band images were obtained on the Chemiluminescent Imaging System (Tanon, 5200, China). All experiments were repeated three times. The density of protein bands was semi quantitatively analyzed by ImageJ software.

### RNA isolation and real-time quantitative polymerase chain reaction (RT-qPCR)

Total RNA of the treated cells was isolated using Trizol reagent (R401, Vazyme, Nanjing, China) per manufacturer's directions, and the RNA concentration was determined by NanoDrop (Thermo Scientific, Rockford, IL). Subsequently, the RNA was converted to cDNA using a reverse transcription kit HiScript II Q RT SuperMix for qPCR (R122-01, Vazyme, China). Next, RT-qPCR was performed using AceQ qPCR SYBR Green Master Mix (Q111-02, Vazyme, China) in a 7500 real-time PCR system (Applied Biosystems, Inc., USA) according to the manufacturer's instruction. The primer sequences used for iNOS, TNF-α, IL-6, IL-1β, and GAPDH are listed in Table S1. The relative expression levels of the target genes were normalized to those of the housekeeping gene GAPDH using the 2ΔΔCt method.

### Enzyme-linked immunosorbent assay (ELISA)

ELISA kits were used to detect inflammatory factors including TNF-α, IL-1β and IL-6 in the injured spinal cord tissue according to the manufacturer's instructions (Mlbio, Shanghai, China). Then, the absorbance at 450 nm was read using a microplate spectrophotometer reader (Tecan, infinite M200 PRO, Austria).

### Statistical analyses

Data are presented as the mean ± standard deviation (SD) for at least three independent biological replicates. After verifying normal distribution and variance homogeneity, one way analysis of variance (ANOVA) was used to analyze the comparison between more than three groups of data followed by Tukey's post hoc analysis, and the comparisons between two groups were performed with the unpaired Student's t test; P-values of < 0.05 were considered statistically significant. All statistical analysis was performed using SPSS Statistics 26.0 software (Chicago, USA).

## Results

### TOF alleviates the severity of secondary injury and improves locomotor function after SCI

**Figure 1:**
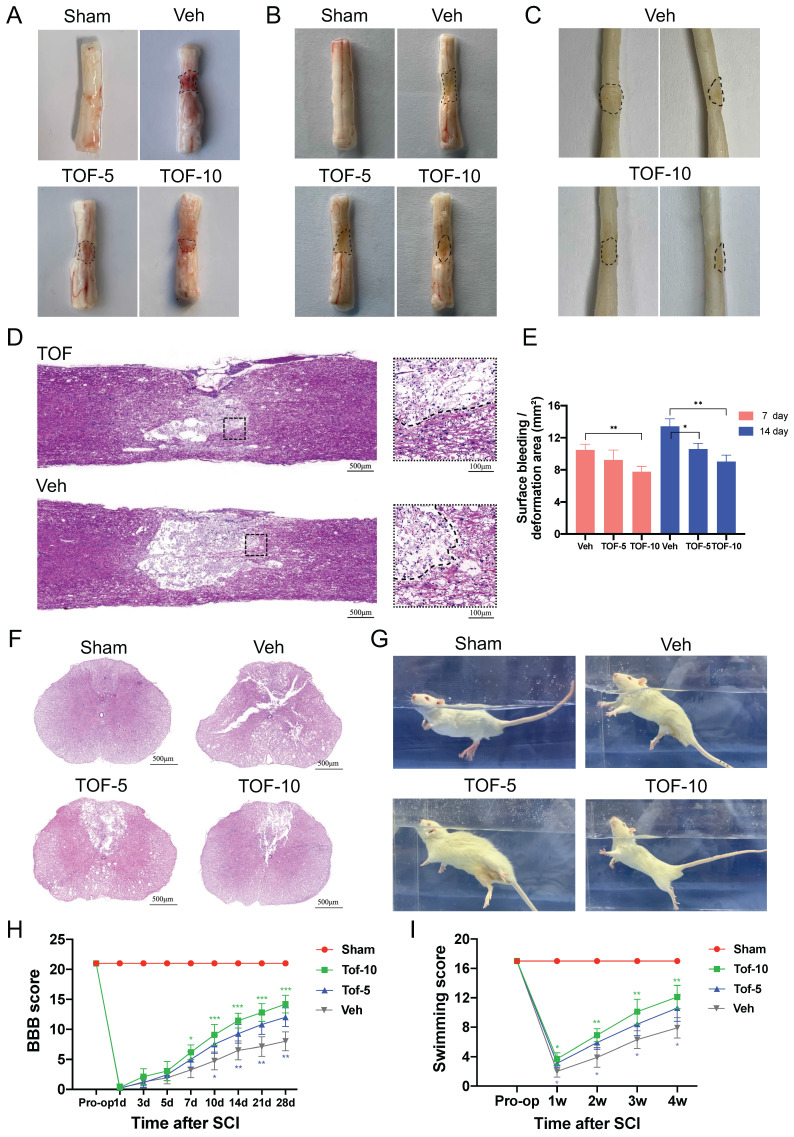
TOF improves pathology and morphology of the spinal cord after injury and promotes locomotor recovery. **(A-C)** Dorsal views of fresh spinal cords on days 7** (A)** and 14 **(B)** post-injury, and dorsal (left) or lateral (right) views of paraformaldehyde-treated spinal cords on day 28 **(C)** post-injury; black dashed lines indicate the lesion areas.** (D, F)** Representative HE-stained longitudinal and transverse sections of the spinal cord on day 28 post-injury. Scale bars = 500 μm. Two small images in **(D)** are the high-magnification view of area around the damage boundary, Scale bars = 100 μm. **(E)** Quantitative analysis of surface bleeding or deformation area in (A).** (G)** Representative images of swimming test in different groups. **(H, I)** The Basso Beattie Bresnahan (BBB) locomotion scores and Louisville Swim scores of different groups over 28-days period (The values are presented as mean ± SD; *p<0.05, **p<0.01, ***p<0.001, one-way ANOVA; n = 8 rats per group).

To evaluate the therapeutic effect of TOF on SCI, the spinal cord tissue was collected at day 5, 14 and 28 after surgery (Figure 1A-C). Compared with Sham group, obvious surface bleeding and hematoma in the early phase of SCI (Figure 1A) and significant cavity and scar formation in the middle phase of SCI (Figure 1B-D) were observed in Veh group. Four weeks after SCI, a widespread loss of the dorsal white matter and central grey matter were observed in the cross sections of the spinal cord in Veh group. After TOF treatment, these pathological changes were improved markedly. The lesion region shrank and more tissue architecture was preserved (Figure 1D, F), and these effects were more pronounced in the high-dose (TOF-10 mg/kg) group. Furthermore, in the process of harvesting the spinal cord tissue, we found an abundant inflammatory fibrous tissue attached around the injured spinal cord, which induced spinal cord compression. In TOP treatment group, the inflammatory infiltration was reduced apparently, and the spinal stenosis and depression caused by the compression were alleviated to some extent.

Knowing that recovery of the motor function after SCI is the main indicator to reflect the outcome of treatment, we assessed it by BBB scale and LSS. As shown in Figure 1H-I, the motor function of rats in each group recovered gradually from day 7 post-SCI, and the recovery in TOF treatment group was significant faster, especially in the high-dose group. The rats treated with TOF exhibited better body balance and motor coordination compared with Veh group at all different time points (Figure 1G).

### TOF alleviates early inflammatory responses after SCI and regulates microglia activation

**Figure 2:**
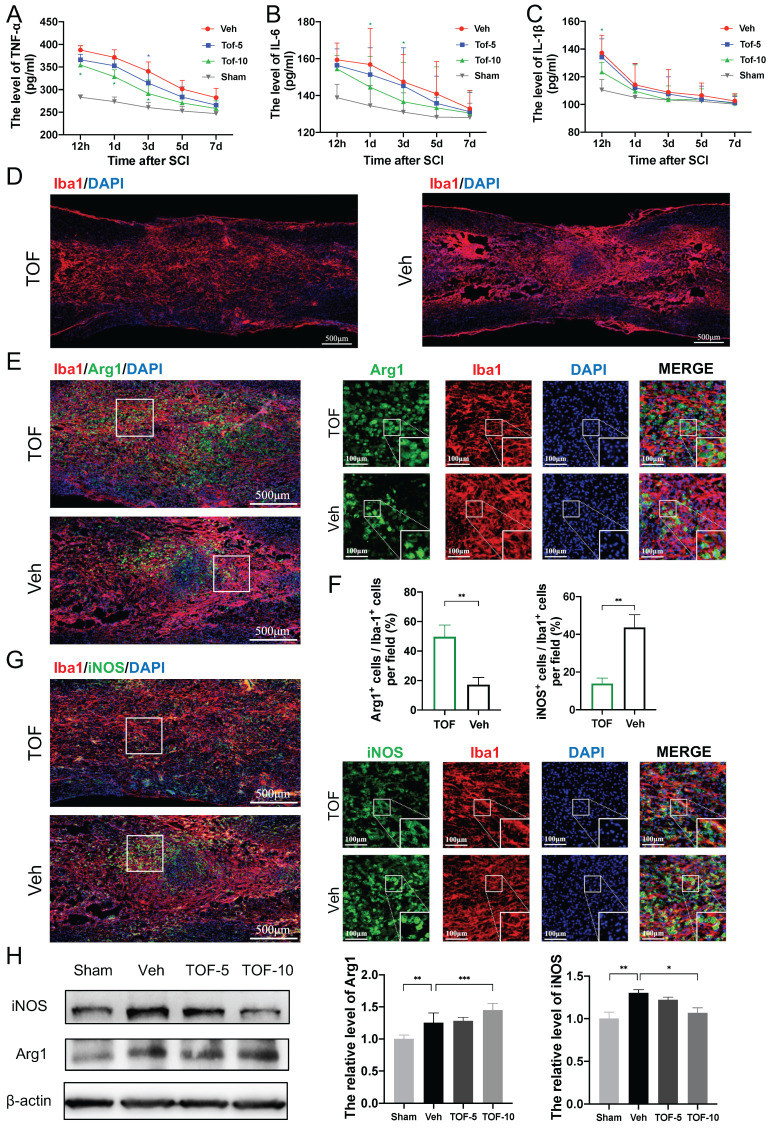
TOF attenuates inflammation by promoting microglia anti-inflammatory polarization and inhibiting pro-inflammatory polarization after SCI. **(A-C)** Concentration-time curves of pro-inflammatory cytokines in spinal cord tissue of each group after SCI. Cytokines in supernatants of tissue lysates were detected by ELISA (The values are presented as mean ± SD; *p<0.05, one-way ANOVA; n = 3 rats per group). **(D)** Representative immunofluorescent images of spinal cord on day 7 post-injury showing the distribution of Iba1 (red) marked microglia in the lesion site. Nuclei were counterstained with DAPI (blue). Scale bar, 500 μm. **(E)** Co-immunofluorescence images showing the anti-inflammatory phenotype microglia labeled by Arg1 (green) and Iba1 (red) in the lesion site on day 7 post-injury. Magnifications of the boxed regions are shown on the right. Scale bar, 500 μm and 100 μm. **(F)** Quantification analysis of the number of Arg1+ Iba1+and iNOS+ Iba1+cells obtained from 3 regions of interest (ROIs) (The values are presented as mean ± SD; **p<0.01, Student's t tests; n = 3). **(G)** Co-immunofluorescence images showing the pro-inflammatory phenotype microglia labeled by iNOS (green) and Iba1 (red) in the lesion site on day 7 post-injury. Magnifications of the boxed regions are shown on the right. Scale bar, 500 μm and 100 μm. **(H)** Western blot analysis and quantification of iNOS and Arg1 expression in spinal cord on day 7 post-injury (The values are presented as mean ± SD; *p<0.05, **p<0.01, ***p<0.001, one-way ANOVA; n = 3 rats per group).

It is well acknowledged that the secondary injury, especially the inflammatory response after SCI, is one of the major factors influencing the survival or apoptosis of neurons in the damaged spinal cord 2. In this study, we detected the levels of pro-inflammatory cytokines in spinal cord supernatants at 12h, 1d, 3d, 5d and 7d after injury by ELISA. In agreement with the previous study 23, it was found in our study that the level of TNF-α, IL-1β and IL-6 protein increased rapidly, peaked at 12 h after SCI, returned nearly to the baseline level within 7 days, and then remained unchanged significantly thereafter (Figure 2A-C). After TOF treatment, especially with high-dose TOF, the level of inflammatory cytokines showed a significant decrease, and soon returned to the normal level. We further explored the cellular mechanism of this anti-inflammatory effect of TOF by immunofluorescence staining to detect the microglial polarization. Under normal physiological conditions, microglia are constantly patrolling the central nervous system. When SCI occurred, microglia migrated to the lesion area rapidly and proliferated profusely, and finally reached the peak between 3-7 days post injury 24. We therefore used 7d after SCI as the time point for microglia evaluation. As indicated in Figure 2D, TOF treatment reduced the accumulation of Iba1 labeled microglia 25 in the lesion area compared with Veh group.

When we identified pro-inflammatory and anti-inflammatory phenotype by co-immunofluorescence staining for iNOS and Arg1 26, more Arg1 positive microglia and fewer iNOS microglia were observed in the injury epicenter in TOF group as compared with Veh group (Figure 2E, G). Correspondingly, the iNOS and Arg1 proteins levels determined by Western blot were significantly higher in SCI lesions. TOF treatment decreased the expression of iNOS, and conversely increased the expression of Arg1 (Figure 2H). Collectively, it is reasonable to believe that TOF could inhibit the inflammatory responses after SCI by promoting microglial switch to anti-inflammatory polarization rather than pro-inflammatory polarization.

### TOF inhibits glial scar formation and promotes neuronal repair

**Figure 3:**
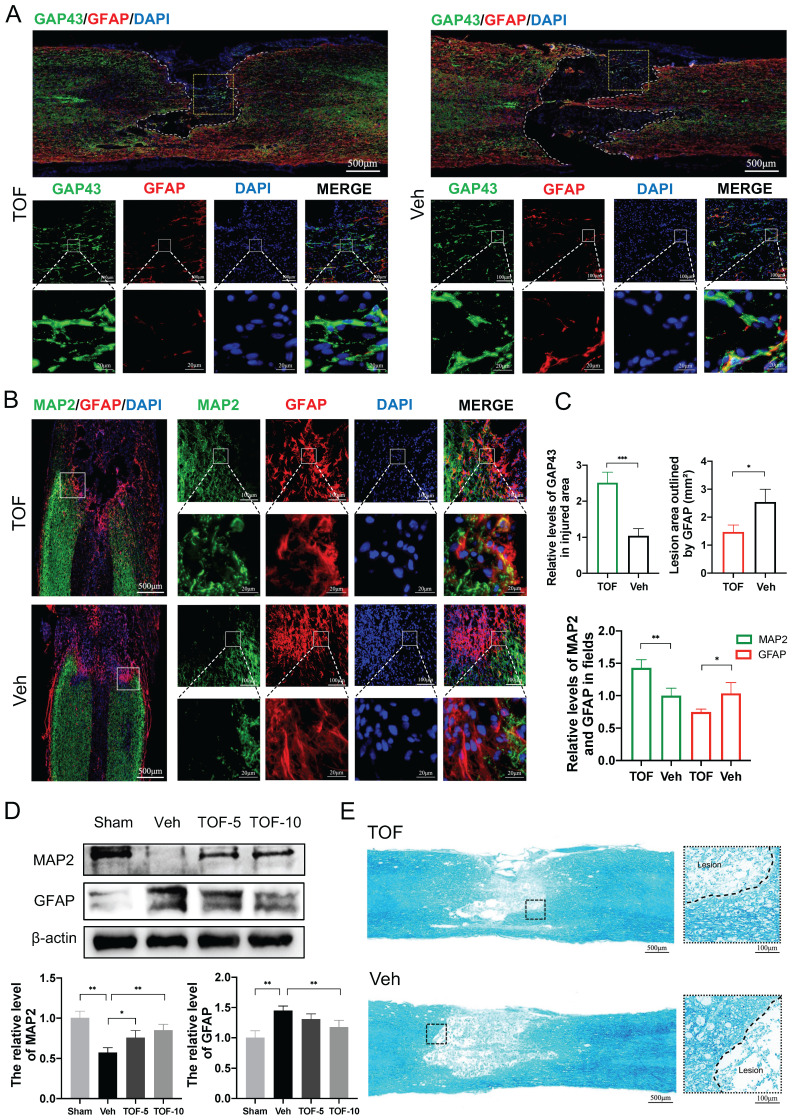
TOF promotes neural structure regeneration and inhibits glial scar formation after SCI. **(A)** Representative images of co-immunofluorescence staining for GAP43 (green) and GFAP (red) on day 28 post-injury in spinal cord lesion areas. TOP row: White dashed lines indicate the lesion border, and yellow boxes indicate the area of axonal structures in the lesion epicenter. Middle and bottom row: Magnifications of boxed region in the upper row. Nuclei were counterstained with DAPI (blue). Scale bar, 500 μm (top), 100 μm (middle) and 20 μm (bottom). **(B)** Representative images of MAP2 (green) and GFAP (red) immunohistochemical staining on day 14 post-injury in the lesion site. Left: White boxes show the junctional region between lesion area and normal area. Right: Magnifications of the boxed regions. Nuclei were counterstained with DAPI (blue). Scale bar, 500 μm (left), 100 μm and 20 μm (right). **(C)** Quantification of the fluorescence intensity and lesion area on day 28 (top) and 14 (bottom) post-injury. (The values are presented as mean ± SD; *p<0.05, **p<0.01, ***p<0.001, Student's t tests; n = 3 per group). **(D)** Western blot analysis and quantification of MAP2 and GFAP expression in spinal cord on day 14 post-injury (The values are presented as mean ± SD; *p<0.05, **p<0.01, one-way ANOVA; n = 3 rats per group).** (E)** LFB staining of transverse section on day 28 post-injury. High-magnification view of area around the damage boundary are shown on the right. Scale bar, 500 μm (left), 100 μm (right).

During SCI repair, axonal rehabilitation is related the recovery of motor function, and glial scar formation is among the major factors hampering regeneration of the nerve tissue. While glial scar limits the lesion and spread of inflammation, it also inhibits axonal regeneration and remyelination 27, 28. In this study, we immune-stained the spinal cord sections with glial fibrillary acidic protein (GFAP) to label astrocytes and glial scar, microtubule-associated protein 2 (MAP2) to label neuronal dendrites 29, growth-associated protein 43 (GAP43) to label axon to evaluate nerve tissue regeneration 30, and used LFB staining to evaluate demyelination. Double immunofluorescence staining in Figure 3A-B showed significant loss of neurons in the lesion region after SCI, especially inside the glial scar, and TOF treatment reduced the lesion area and glial scar significantly, as shown by more green-labeled axonal structures and LFB-stained myelinated axons in the injury epicenter at day 28 after SCI (Figure 3A, E). During the early maturation stage of astroglial scarring at day 14 after injury 31, more MAP2 positive neurofibrils penetrating the glial scar were observed in TOF group, while the astroglial scar in Veh group seemed to completely block the growth of the neurofibrils (Figure 3B). Besides, the density of red-labeled astroglial scar in TOF group was notably lower than that in Veh group on the locally enlarged images. Western blot showed that he MAP2 protein level was upregulated and the GFAP protein level was down-regulated in the injured spinal cord tissue in TOF group, and this effect was more pronounced in high-dose TOF group (Figure 3D). Taken together, these data demonstrated that TOF could potently inhibit glial scar formation and promote the survival or regeneration of neuron structures such as axons, dendrites, and myelin.

### TOF inhibits neuronal apoptosis after SCI

**Figure 4:**
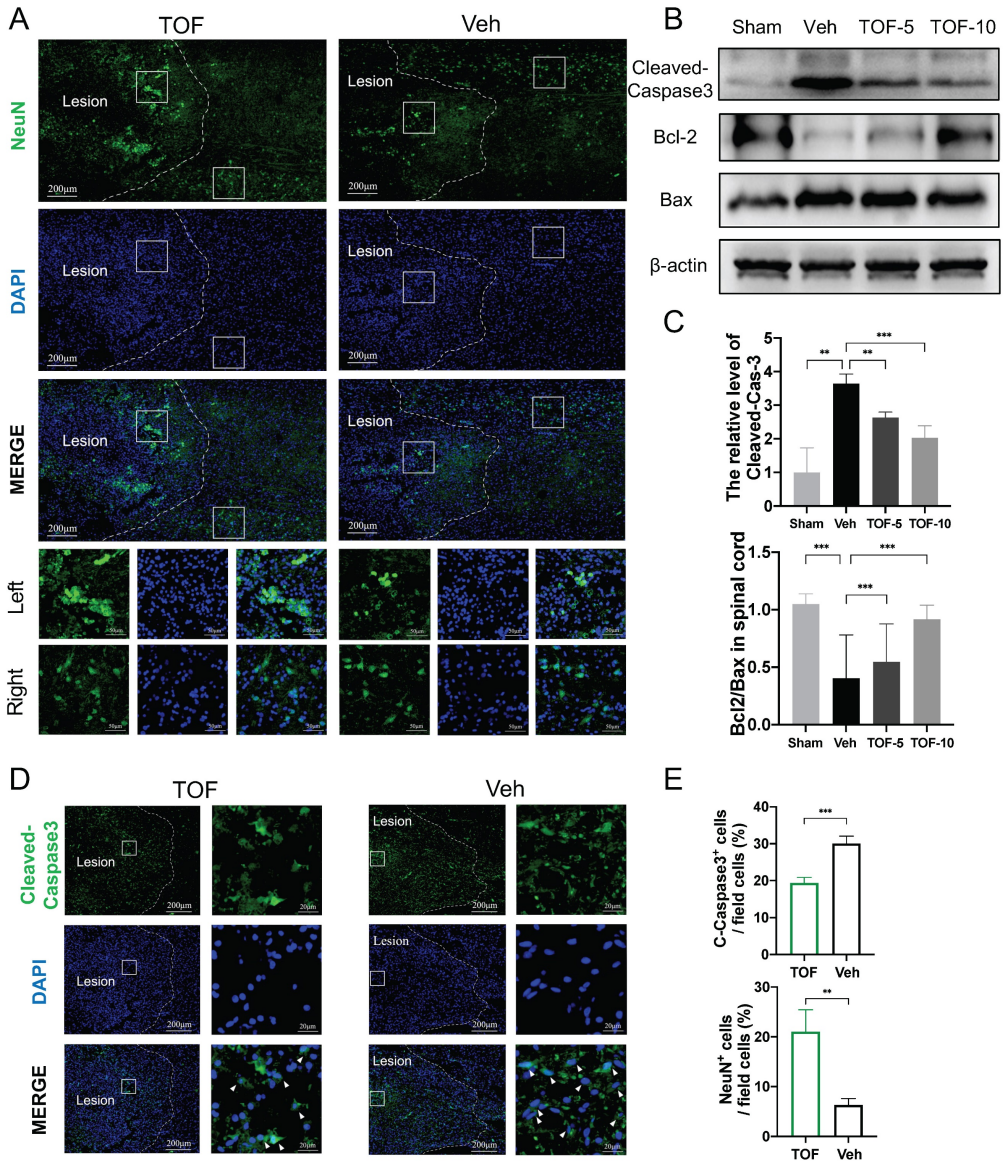
TOF promotes neuron survival and ameliorates neuronal apoptosis after SCI. **(A)** Immunofluorescent images of NeuN (green) showing the arrangement of neurons on day 14 post-injury. The boxed areas represent representative images of injured (left) and uninjured (right) area. Two bottom rows are the enlarged images of the injured and uninjured area in different group. Nuclei were counterstained with DAPI (blue). Scale bar, 200 μm and 50 μm. **(B)** Western blot analysis performed for cleaved-caspase3, Bcl-2 and Bax expression in spinal cord obtained on day 14 post-injury. **(C)** Quantification of cleaved-caspase3 protein expression level and the ratio of Bcl-2/Bax (The values are presented as mean ± SD; **p<0.01, ***p<0.001, one-way ANOVA; n = 3 rats per group). **(D)** Immunofluorescent images of the apoptosis marker Cleaved-Caspase3 (green) in the damage area on day 14 post-injury. Magnifications of the boxed regions are shown on the right. Nuclei were counterstained with DAPI (blue). Scale bar, 200 μm and 20 μm. **(E)** Quantification analysis of the number of NeuN+ and Cleaved-Caspase3+ cells obtained from 3 ROIs (The values are presented as mean ± SD; **p<0.01, ***p<0.001, Student's t tests; n = 3).

To observe the apoptosis of neurons in the middle phase of SCI, immunofluorescence for Neuron (NeuN) and Cleaved-Caspase3 staining was performed on longitudinal sections of the spinal cord. As demonstrated in Figure 4A, on the longitudinal section, the neurons in the undamaged area were arranged neatly and regularly. In contrast, impact injury of the spinal cord resulted in a reduction of neurons and overlapping aggregation of neurons in the damaged area. In TOF group, the number of neurons was increased to a certain extent (Figure 4A, E), and Cleaved-Caspase3-positive cells were significantly decreased on the local enlarged image of the lesion area in comparison with Veh group (Figure 4D-E). The similar results were confirmed by Western blot analysis, showing that the elevated expression of activated caspase3 was normalized and the Bcl2/Bax ratio as a crucial determinant of apoptosis was decreased in the central lesion following SCI after TOF adminitration (Figure 4B-C). All the above findings demonstrated that TOF had a protective effect on neurons and inhibited apoptosis caused by SCI in a rat model.

### TOF inhibits LPS-induced pro-inflammatory activation of microglia *in vitro*

To further confirm whether TOF affects microglial polarization *in vitro*, we analyzed the effect of different doses of TOF on LPS-induced pro-inflammatory polarization of primary microglia and BV2 cells. Firstly, the toxicity of TOF on different cells was examined by CCK8 assay, showing that TOF had no toxic effect at concentrations up to 100μM (Figure S1). We therefore chose 30μM, 60μM and 90μM as the concentrations of TOF for subsequent experiments. It was reported that under normal condition, microglial displayed the typical ramified morphology. Following injury *in vivo* or activated by the stimulation of LPS *in vitro*, microglial adopt an amoeboid shape which are characterized by shortened branches 32. It was found in our study that the morphological change triggered by LPS was reversed by TOF pretreatment (Figure S2A).

**Figure 5:**
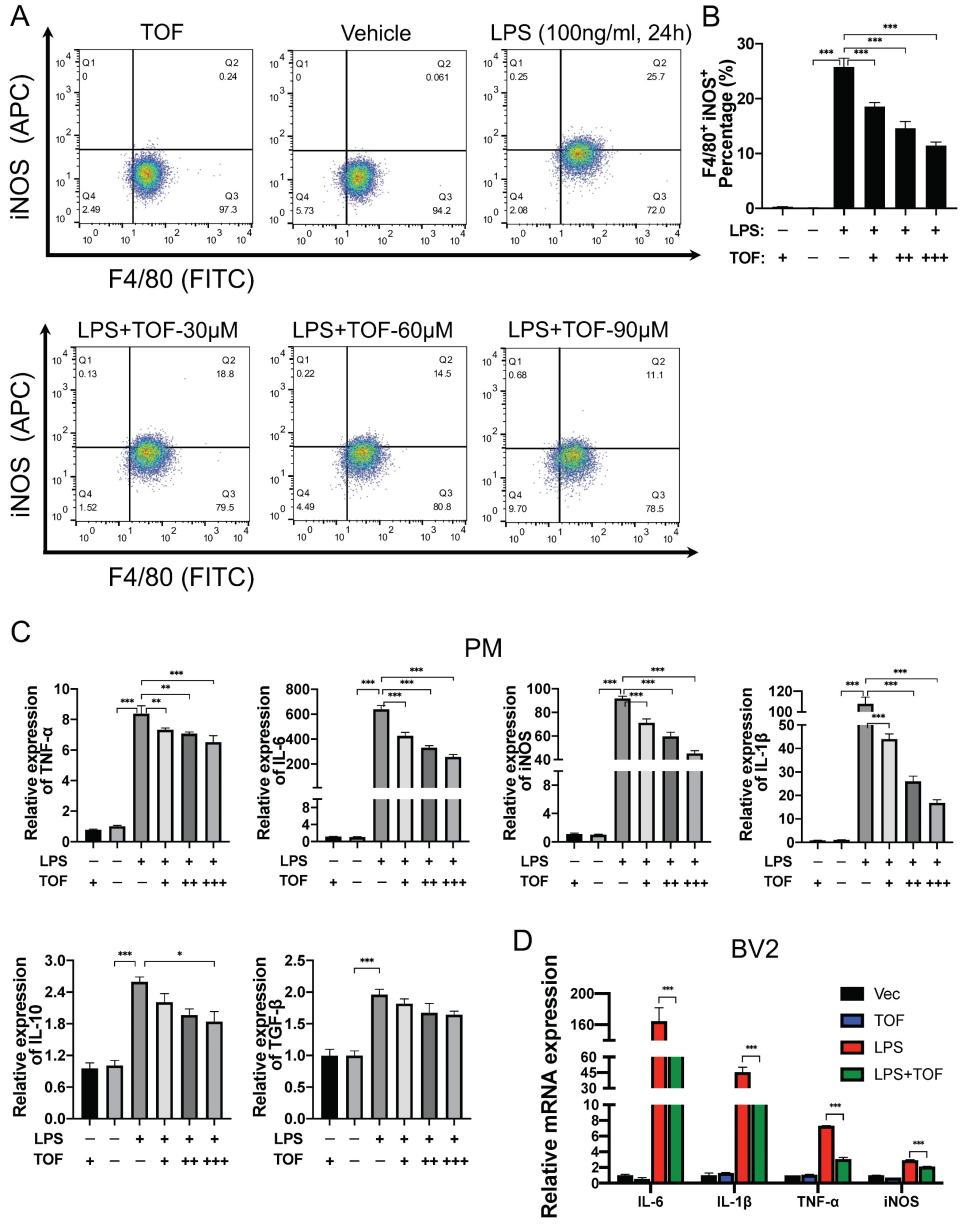
TOF suppresses microglia polarization toward pro-inflammatory phenotype *in vitro*. **(A-B)** Representative flow cytometric analysis of LPS-induced primary microglia in each group. Histogram showing the calculated percentage of pro-inflammatory phenotype (iNOS+/Iba1+) primary microglia (The values are presented as mean ± SD; **p<0.01, ***p<0.001, one-way ANOVA; data are combined from 3 independent experiments). **(C-D)** RT-qPCR analysis of pro-inflammatory related genes expression in primary microglia (PM) and BV2 cells. All data were normalized to GAPDH expression (The values are presented as mean ± SD; *p<0.05, **p<0.01, ***p<0.001, one-way ANOVA; data are combined from 3 independent experiments).

**Figure 6:**
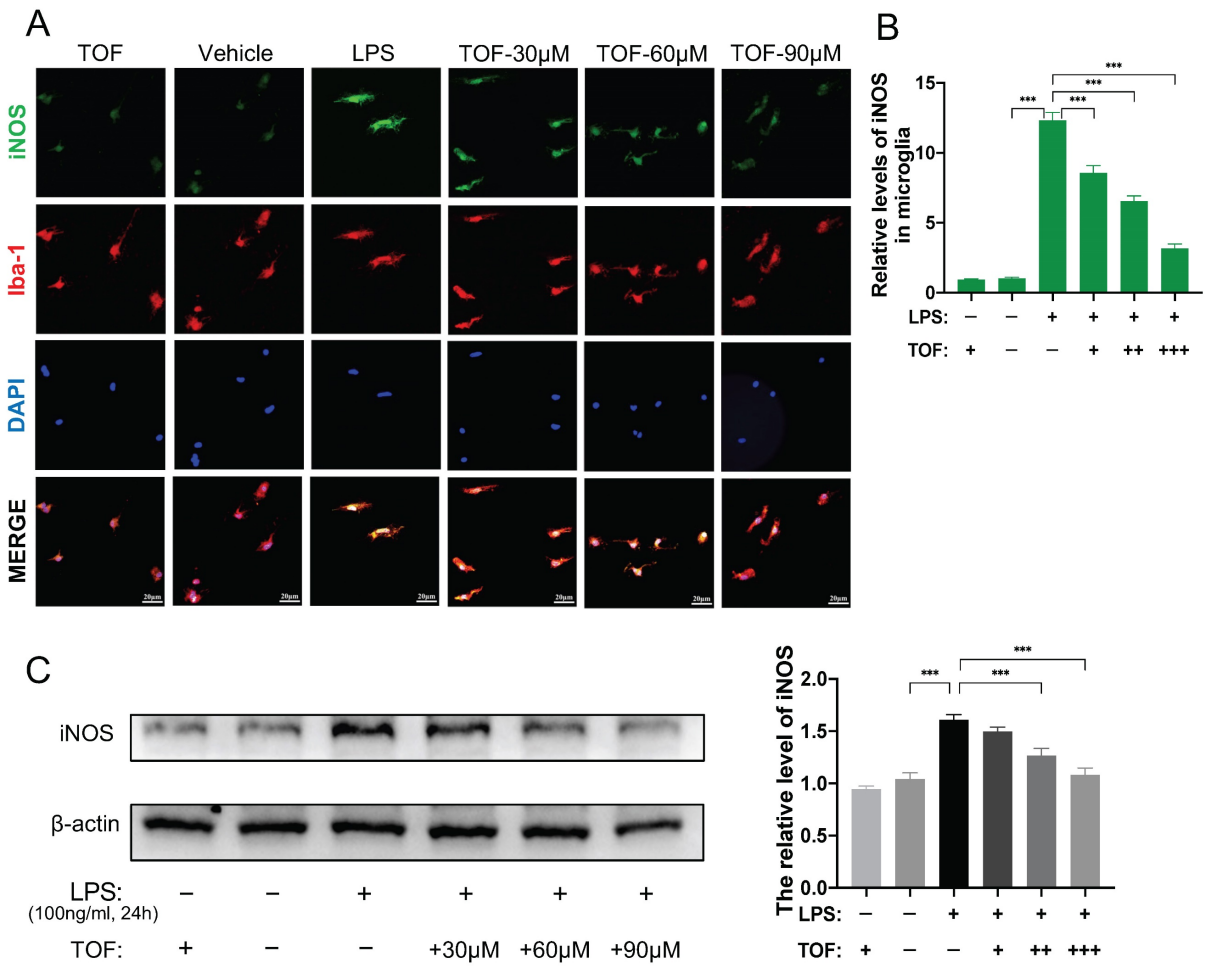
TOF suppresses microglia polarization toward pro-inflammatory phenotype *in vitro*. **(A)** Representative immunofluorescent staining of iNOS (green) and Iba1 (red) in primary microglia in each group. Nuclei were counterstained with DAPI (blue). Scale bar, 20 μm. **(B)** Quantification of the iNOS fluorescence intensity. (The values are presented as mean ± SD; *p<0.05, **p<0.01, ***p<0.001, Student's t tests; data are combined from 3 independent experiments). **(C)** Western blot analysis and quantification of iNOS expression in primary microglia (The values are presented as mean ± SD; *p<0.05, **p<0.01, one-way ANOVA; data are combined from 3 independent experiments).

We further used flow cytometry analysis to identify the effect of TOF on LPS-induced microglia polarization. As displayed in Figure 5A-B, TOF treatment significantly reduced the percentage of iNOS and F4/80 positive cells in primary microglia. Similar results were also obtained in BV2 cells (Figure S2B-C). In terms of protein and mRNA levels, we detected the expression of pro-inflammatory genes such as iNOS, IL-6, TNF-α, IL-1β, and anti-inflammatory genes such as IL-10 and TGF-β. TOF pretreatment reversed the increase in the expression of pro-inflammatory markers in both primary microglia and BV2 cells after LPS induction, and exhibited a similar suppressive effect on pro-inflammatory gene expression in uninduced cells in Control group as well (Figure 5C-D, Figure 6C, Figure S3). On the other hand, TOF pretreatment also appeared to slightly inhibit the expression of anti-inflammatory genes, although this effect was not significant. Similar results were also obtained using immunofluorescence, showing that the fluorescence intensity of iNOS was significantly increased after LPS stimulation, which was reversed by TOF pretreatment in a dose-dependent manner (Figure 6A-B, Figure S2D). Although in our study, the cell culture system was used to induce pro-inflammatory polarization, when we examined the Arg1 level associated with anti-inflammatory polarization, TOF reversed the decline in its expression (Figure S3). In summary, these results indicated that TOF could inhibit pro-inflammatory polarization of microglial *in vitro*, and this phenomenon may correlate with the promotion of anti-inflammatory polarization.

### TOF protects neuronal cells against stimulation of activated microglia *in vitro*

**Figure 7:**
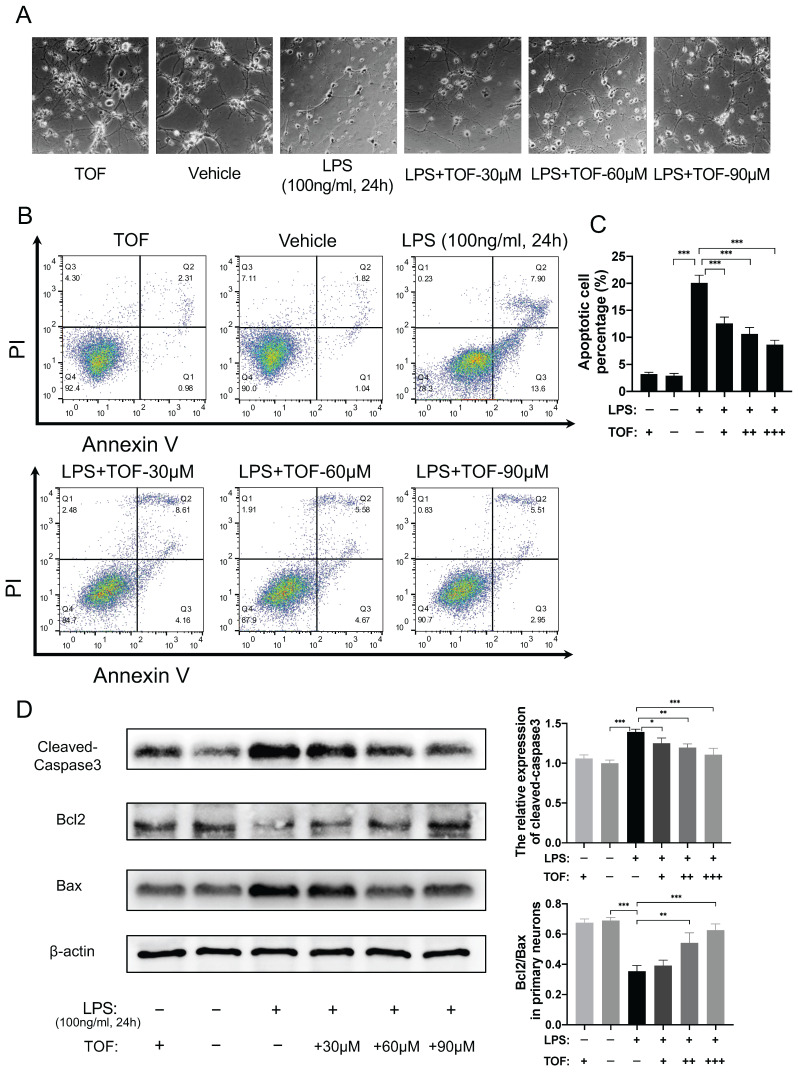
TOF inhibits activation of microglia-induced neuronal apoptosis* in vitro*. **(A)** The morphology of primary neurons after co-culture with active microglia. **(B-C)** Flow cytometry analysis of primary neuron apoptosis in each group. Histogram showing the calculated percentage of apoptotic cell marked with Annexin V+ (The values are presented as mean ± SD; ***p<0.001, one-way ANOVA; data are combined from 3 independent experiments). **(D)** Western blot analysis and quantification of cleaved-caspase3, Bcl-2 and Bax expression in primary neurons (The values are presented as mean ± SD; * p<0.05, **p<0.01, ***p<0.001, one-way ANOVA; data are combined from 3 independent experiments).

Activated microglia can mediate neuroinflammation via releasing reactive oxygen species (ROS) and pro-inflammatory cytokines, leading to neuronal apoptosis, neuroexcitotoxicity, and necrotic death 33, 34. To determine whether TOF could protect neuronal cells under inflammatory conditions, we co-cultured primary neurons or HT22 cells with LPS-induced BV2 cells. Morphologically, normal neuron cells are spherical with many neurites. When they were co-cultured with activated microglia, both primary neurons and HT22 cells exhibited neuronal soma atrophy and neurite retraction as previously reported in the literature 35 (Figure 7A, Figure S4A). In contrast, pretreatment of neurons with TOF before the co-culture partially restored their normal morphology. Meanwhile, we tested the protection role of TOF in cell survival using Annexin V- FITC/PI staining. As shown in Figure 7B-C, the proportion of apoptotic neurons in LPS-induced group was significantly higher than that in vehicle group (without LPS), whereas TOF could decrease this proportion in a dose-dependent manner. In addition, the expression of apoptosis-related proteins was reduced by TOF treatment as well, where the Bcl2/Bax ratio and the activated caspase3 level were reduced compared with those in LPS group (Figure 7D). Similar results were also obtained using HT22 cells (Figure S4), especially when only adherent cells were collected after co-culture, TOF significantly reduced the proportion of early apoptotic HT22 cells (Annexin V+/PI -). It is worth mentioning that cleaved-caspase3 is also a marker of early apoptosis, and the change of its expression level is completely consistent with the results of flow cytometry. These data indicate that TOF exerted its protective effect on neurons co-cultured with pro-inflammatory phenotype microglia.

### STAT signaling was activated in the injured spinal cord and *ex vivo* microglia in inflammatory states, which could be effectively inhibited by TOF administration

**Figure 8:**
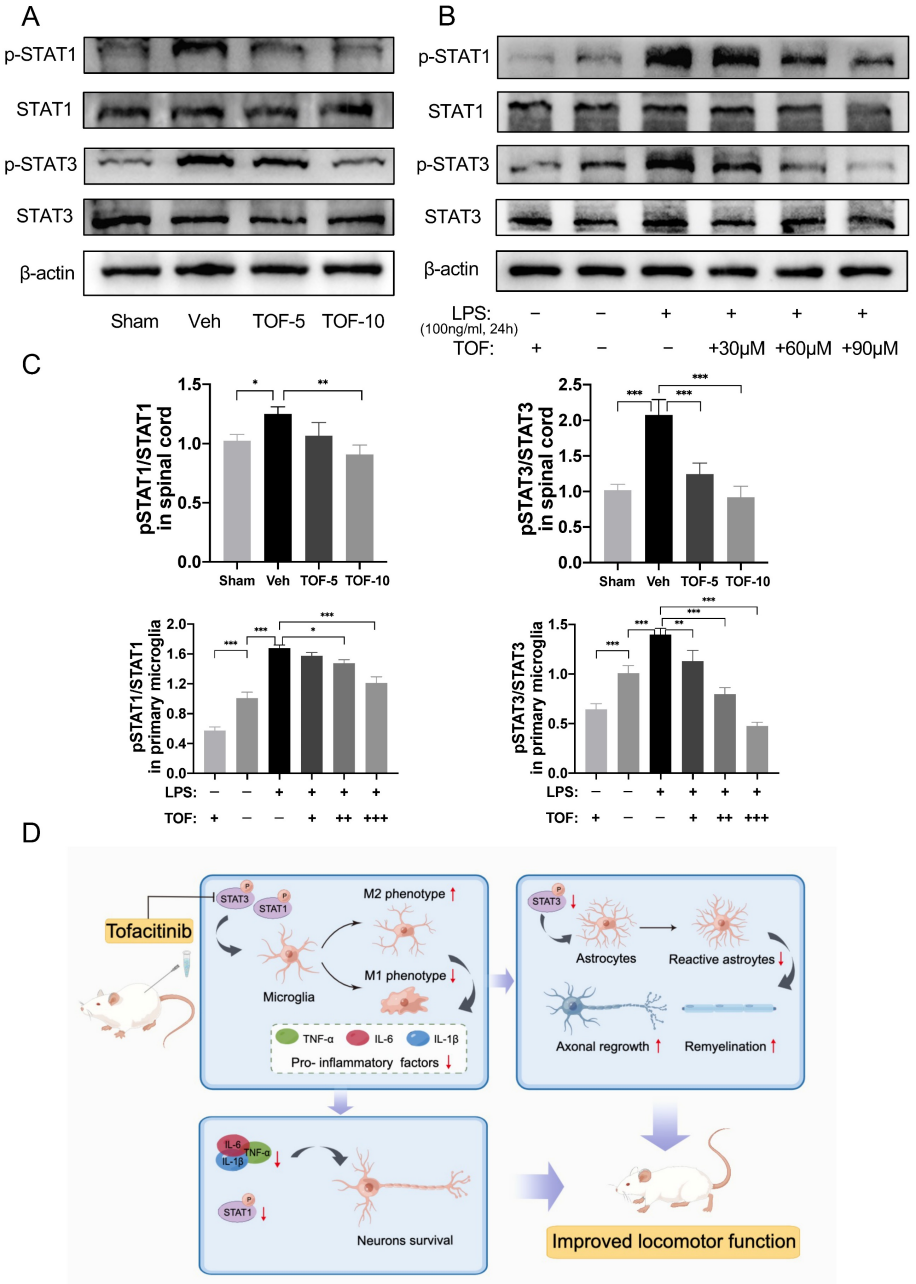
TOF normalizes the activated STAT signaling in injured spinal cord and *ex vivo* microglia under inflammatory conditions. **(A)** Western blot analysis performed for p-STAT1, STAT1, p-STAT3 and STAT3 expression in spinal cord obtained on day 7 post-injury. **(B)** Western blot analysis of p-STAT1, STAT1, p-STAT3 and STAT3 expression in primary microglia. **(C)** Quantification of the ratio of p-STAT1/ STAT1 and p-STAT3/ STAT3 (The values are presented as mean ± SD; *p<0.05, **p<0.01, ***p<0.001, one-way ANOVA; n = 3 rats per group or combined from 3 independent experiments). **(D)** Proposed mechanism: TOF regulates inflammation and promotes neural repair after SCI.

Many studies have reported activation of the JAK/STAT signaling pathway in the process of spinal cord damage repair after SCI 11, 36, 37. Specifically, STAT1 or STAT3 was dominantly activated according to the severity of SCI 36, 37. It was found in our study that the phosphorylation of STAT1 and STAT3 was significantly increased in the injured spinal cord tissue at the early stage of injury, and both were effectively reduced after TOF treatment in a dose-dependent manner (Figure 8A, C). Similar results were confirmed *in vitro*, where the expression of phosphorylated STAT1 and phosphorylated STAT3 was increased significantly in LPS-induced microglia (Figure 8B-C, Figure S3A, C). After TOF treatment, STAT1 and STAT3 phosphorylation was decreased whether under the normal culture medium or LPS stimulation. These results demonstrate that TOF treatment could indeed inhibit STAT signaling through the JAK/STAT pathway both in tissues and cells, which in turn affected microglial polarization.

## Discussion

Unlike primary irreversible injury, secondary injury is a reversible pathological process. How to intervene and alleviate the secondary injury, especially to inhibit the cascade of inflammation, is the key to the treatment of SCI. Therefore, the inhibitory effect of TOF on inflammation may be of great value for early treatment of SCI. Motivated by this perspective, this study for the first time reported the therapeutic effect of TOF on SCI, and explored the therapeutic mechanism of TOF in inhibiting the inflammatory response of secondary injury by affecting microglial activation.

The recovery of secondary SCI depends on the mutual regulation of microglia, astrocytes and neurons through cytokines, which is ultimately manifested as a long inflammatory repair process in pathology 2. Numerous studies have demonstrated that the JAK/STAT signaling pathway is activated after SCI, which plays different roles in regulation of above nerve cells. In the acute phase after SCI (≤48h), p-STAT3 was highly expressed in injured neurons, while in the subacute phase, p-STAT3 was mainly present in activated astrocytes and microglia 11. In addition, conditional knockout of STAT3 in astrocytes could inhibit astrocyte proliferation after SCI, ultimately leading to the interruption of glial scar formation 38. Osuka et al. 36 studied the spatiotemporal patterns of STAT1 expression after SCI and found that the activation of STAT1 may trigger neuronal apoptosis. Based on their finding, further study demonstrated that selective inhibition of STAT1 could alleviate SCI in mice 37.

However, scholars often used different cognitions of JAK/STAT pathway according to different research objects, suggesting that this pathway plays different roles in different stages of disease evolution. On the one hand, Yamauchi et al. 11 considered that STAT3 activation induced by IL-6 contributed to the protection of neurons after SCI. Park et al. 39, 40 also found that the elevated expression of endogenous SOCS3 in spinal cord neurons after SCI could lead to decreased mitochondrial STAT3 level, which would eventually result in neuronal death. On the other hand, with the deepening of research on the pathological mechanisms of SCI, increasing numbers of scholars believe that the activation of the immune system after SCI is one of the main pathophysiological triggers for tissue destruction 41, and neuroinflammation is a crucial factor affecting neuronal apoptosis 42.

Meanwhile, the immune system plays a synergistic role in nerve regeneration after SCI 43. Thus, many researchers consider that the regulation of inflammatory responses via JAK/STAT pathway may affect the prognosis of SCI, and get some evidence from the study of macrophage/microglia polarization. As introduced earlier, the proportion of pro- and anti-inflammatory phenotype of macrophage/microglia modulated the outcome of SCI. Under hypoxic conditions, oxidative stress-induced STAT1 phosphorylation could activate pro-inflammatory polarization of microglia and neuroinflammation 13. While with the treatment of anti-STAT1 polyphenol myricetin, microglia activation was suppressed, which counteracted neuronal death 14. In addition, the inhibition of p-STAT1 and SOCS3 was shown to be able to promote the anti-inflammatory polarization of macrophages following SCI on the one hand, and suppress the secretion of IL-6 by pro-inflammatory phenotype macrophages on the other hand 44. Scholars have also demonstrated the therapeutic mechanism of some drugs for SCI, which may be related to the decreased phosphorylation and increased acetylation of STAT1 7, 45.

As a small molecule JAK inhibitor, TOF mainly competitively inhibits the phosphorylation of JAK kinases 1 and 3, thereby blocking the recruitment and phosphorylation of downstream molecules, resulting in the inability of STATs to enter the nucleus to initiate the transcription of inflammation-related genes 19. In this study, we demonstrated that STAT1 and STAT3 were activated in the spinal cord tissue after SCI as previously reported. After TOF treatment, the expression of p-STAT1 and p-STAT3 was significantly inhibited, which in turn decreased the levels of inflammatory factors such as IL-6, TNF-α, IL-1β in the injured spinal cord, and reduced the recruitment of microglia in the injured area. Meanwhile, this anti-inflammatory action was also manifested in microglial polarization, i.e. TOF induced the transformation of microglia from pro-inflammatory phenotype to anti-inflammatory phenotype in the lesion area. Immunofluorescence staining showed that TOF inhibited neuronal apoptosis after SCI, which is not only closely related to the anti-inflammatory effect of TOF but also is associated with the roles of STAT1 and STAT3 in the process of SCI. According to Osuka et al. 36, STAT3 was dominantly activated compared with STAT1 and subsequently activated the antiapoptotic genes in mild SCI, while in severe SCI, more STAT1 phosphorylation was induced, leading to cell apoptosis. In addition, we also noted that there were more neurofibril regeneration in the periphery of the glial scar in TOF groups, probably because TOF not only reduced the aggregation of microglia, which is a key cellular component of the scar 46, but also inhibited the activation of STAT3 and impeded the proliferation of reactive astrocytes, thus reducing glial scar formation 31, 38. Although some scholars believe that glial scar prevents expansion of the inflammatory process, which is essential for acute containment of inflammation, the prevailing view proposes that scar tissue formation ultimately leads to the failure of axon regeneration 2. The results of our study suggest that the decreased level of GFAP expression may favor axon regeneration and remyelination. As indicated in several studies, the use of JAK/STAT inhibitors could repress reactive astrogliosis, diminish activation of microglia, promote neuronal survival and neurite outgrowth, and finally alleviate the hyperalgesia after SCI 12, 47. Combining the above results and analysis, TOF-treated rats ultimately exhibited significant recovery of locomotor function after SCI.

At the cellular level, we focused on the effect of TOF on microglia polarization in primary microglia and BV2 cells. Previous studies have shown that LPS can induce inflammatory activation in macrophages through different signaling cascades activated in response to TLR4 activation, including the classical MAPK family and the NF-κB pathway. With further studies, researchers have found that the macrophage response is the result of a complex intracellular signaling network in which the response of STAT3 and its cross-talk with p38MAPK play an important role 48. In addition, IRF/STAT pathway is a central pathway that regulates macrophage polarization, and that activation of IRF/STAT pathway through STAT1 will skew macrophage toward the pro-inflammatory phenotype, and the activation through STAT6 will skew macrophage toward the anti-inflammatory phenotype 49, while the role of STAT3 (SOCS3) in it is still controversial 50. In our study, the expression of p-STAT1 and p-STAT3 was significantly increased in LPS-induced pro-inflammatory microglia compared with that in nonactivated cells. TOF treatment inhibited the mRNA expression of the pro-inflammatory polarization markers including iNOS, TNF-α, IL-1β, and IL-6, and increased the Arg1 and CD163 level, which is also supported by other *in vitro* experiments such as bone marrow-derived macrophages 51 and macrophage cell line (RAW264.7) 52. In addition, the effect of TOF on the anti-inflammatory factor TGF-β was not significant, and TOF even exerted a certain inhibitory effect on IL-10, which may be closely related to its blocking of JAK1 and TYK2 53. Interestingly, some scholars also observed different results. Lescoat et al. 54 found that TOF inhibited both M1 and M2 polarization of macrophages in both* in vivo* and* in vitro* models of fibrotic diseases. Stalder et al. 55 even obtained the puzzling results that TOF promoted M1 polarization. In our opinion, this discrepancy in results might be derived from the differences in research methods, especially the way of polarization induction. Furthermore, macrophages play different roles in different disease models, which can also bias the findings.

To the best of our knowledge, this is the first study clarifying the effect of TOF on microglial polarization and neuroprotection. However, more research needs to be done to explain how TOF affects the complex pathological process of SCI, as well as the specific effects on other cells. In addition, by comparing TOF with other JAK inhibitors, some investigators found that the potential of TOF to affect macrophage activation and polarization depended on the activity of JAK1 rather than JAK3 50, suggesting that there may be better JAK/STAT pathway inhibitors available for the treatment of SCI.

## Conclusions

Our study demonstrated that TOF ameliorated inflammatory responses after SCI by affecting the activation of microglia via JAK/STAT signaling pathway. This anti-inflammatory effect of TOF promoted neural structure regeneration and reduced neuronal apoptosis, ultimately improving motor function in rats post injury. Based on our data both* in vivo* and *in vitro*, it is reasonable to believe that TOF treatment could be a potential therapeutic strategy for SCI and our findings will be valuable for the early treatment of SCI.

## Supplementary Material

Supplementary figures and table.

## Abbreviations

Arg1: Arginase 1
Bax: Bcl2-associated X protein
BBB: Basso-Beattie-Bresnahan
Bcl-2: B-cell lymphoma 2
CCK-8: Cell Counting Kit-8
ELISA: Enzyme-linked immunosorbent assay
GAP43: Growth-associated protein 43
GFAP: Glial fibrillary acidic protein
Iba1: Ionized calcium binding adaptor molecule 1
IL: Interleukin
iNOS: Inducible nitric oxide synthase
JAK: Janus kinase
LFB: Luxol fast blue
LSS: Louisville Swim Score
MAP2: microtubule-associated protein 2
PM: Primary microglia
ROI: Regions of interest
SCI: Spinal cord injury
STAT: Signal transducers and activators of transcription
TNF: Tumor necrosis factor
TOF: Tofacitinib
