# Supplementary material for: Tofacitinib Promotes Functional Recovery after Spinal Cord Injury by Regulating Microglial Polarization via JAK/STAT Signaling Pathway
Source: Int J Biol Sci. 2023 Sep 11;19(15):4865–82. doi: 10.7150/ijbs.84564 (PMC10539697; doi:10.7150/ijbs.84564)
Supplement: Supplementary file 1 — Supplementary figures and table. [file ijbsv19p4865s1.pdf]

Supplementary information

TABLE S1 List of primers used for RT-qPCR.

Supplementary Table 1: The primer sequences used for qPCR		
Genes	Forwards (5'to3')	Reverse (5'to3')
**Mouse- Tnfα**	CCCTCACACTCAGATCATCTTCT	GCTACGACGTGGGCTACAG
**Mouse- iNOS**	GTTCTCAGCCCAACAATACAAGA	GTGGACGGGTCGATGTCAC
**Mouse- IL-6**	TAGTCCTTCCTACCCCAATTTCC	TAGTCCTTCCTACCCCAATTTCC
**Mouse-IL1β**	TGGAAAAGCGGTTTGTCTTC	TACCAGTTGGGGAACTCTGC
**Mouse- Arg1**	GTGAAGAACCCACGGTCTGT	GCCAGAGATGCTTCCAACTG
**Mouse-CD163**	GCCATAACTGCAGGCACAAA	GTTGGTCAGCCTCAGAGACA
**Mouse-GAPDH**	AGGTCGGTGTGAACGGATTTG	GGGGTCGTTGATGGCAACA

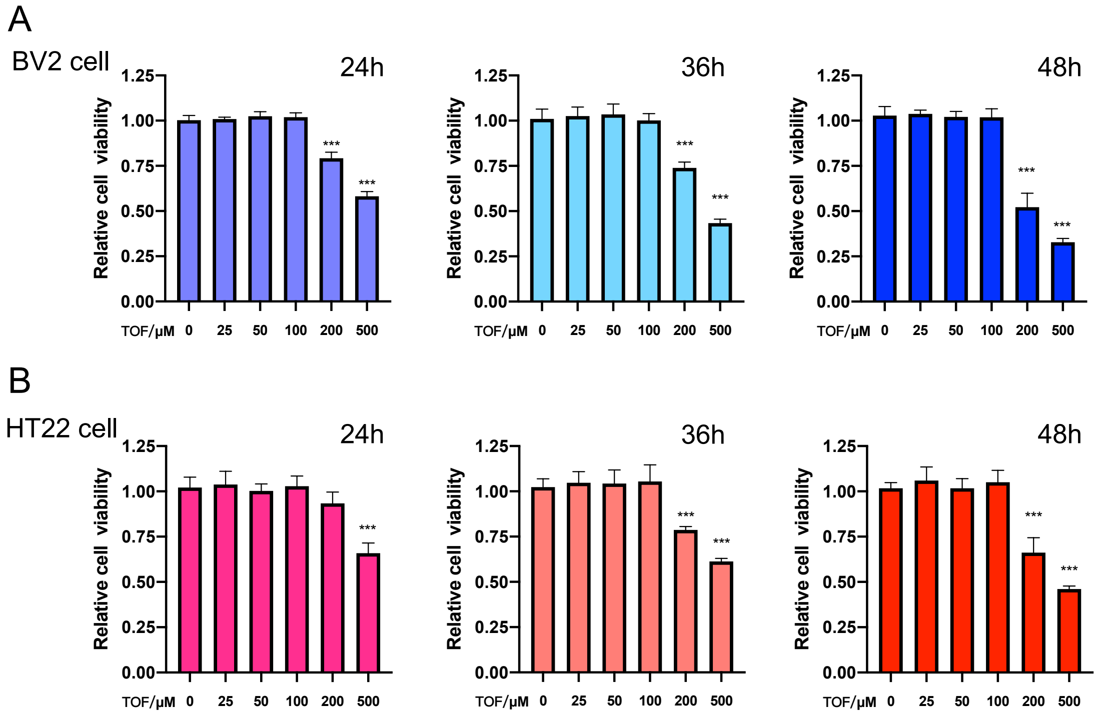

Figure S1. (A-B) Cell viability analysis using the CCK-8 assay in BV2 cells and HT22 cells (The values are presented as mean ± SD; ***p<0.001, one-way ANOVA; n = 5 per group).

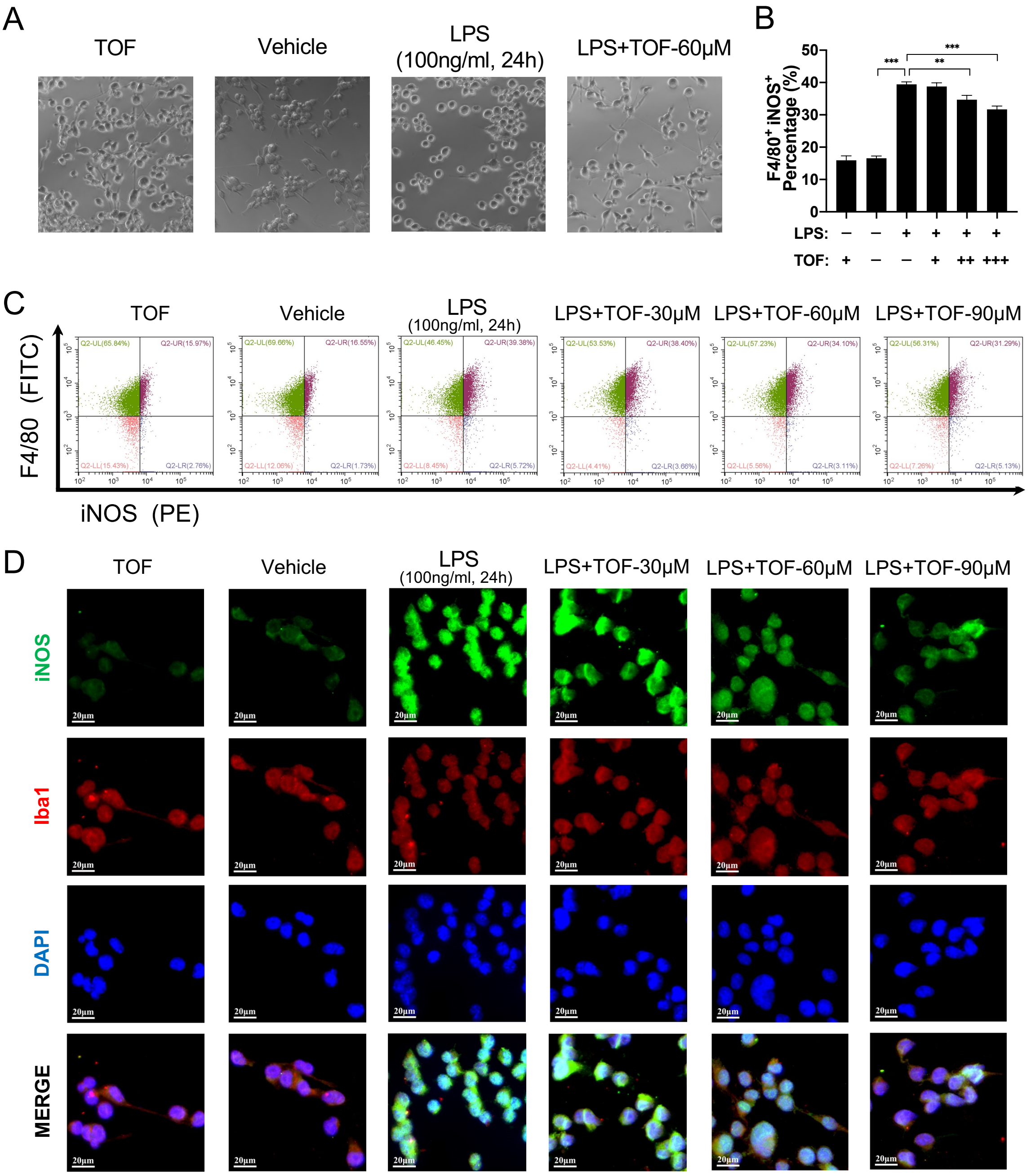

Figure S2. (A) The morphology of BV2 cells after different treatment. **(B-C)** Representative flow cytometric analysis of LPS-induced BV2 cells in each group (The values are presented as mean ± SD; **p<0.01, ***p<0.001, one-way ANOVA). **(D)** Representative immunofluorescent staining of iNOS (green) and IBA-1 (red) in BV2 cells in each group. Nuclei were counterstained with DAPI (blue). Scale bar, 20 μm.

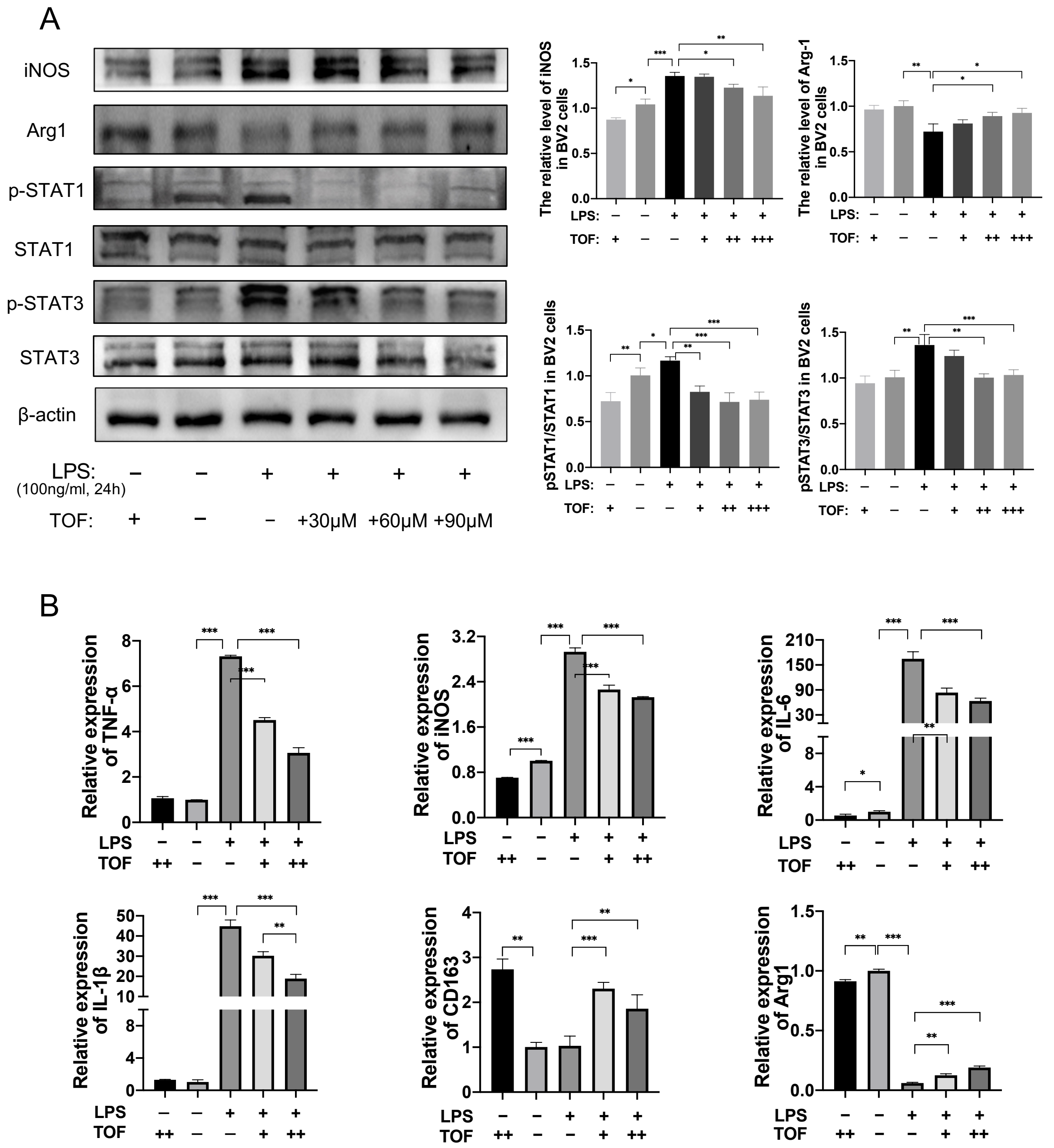

Figure S3. (A) Western blot analysis and quantification in BV2 cells (The values are presented as mean ± SD; **p*<0.05, ***p*<0.01, ****p*<0.001, one-way ANOVA). **(B)** RT-qPCR analysis of pro-inflammatory and anti-inflammatory related genes expression in BV2 cells. All data were normalized to GAPDH expression (The values are presented as mean ± SD; *p<0.05, **p<0.01, ***p<0.001, one-way ANOVA).

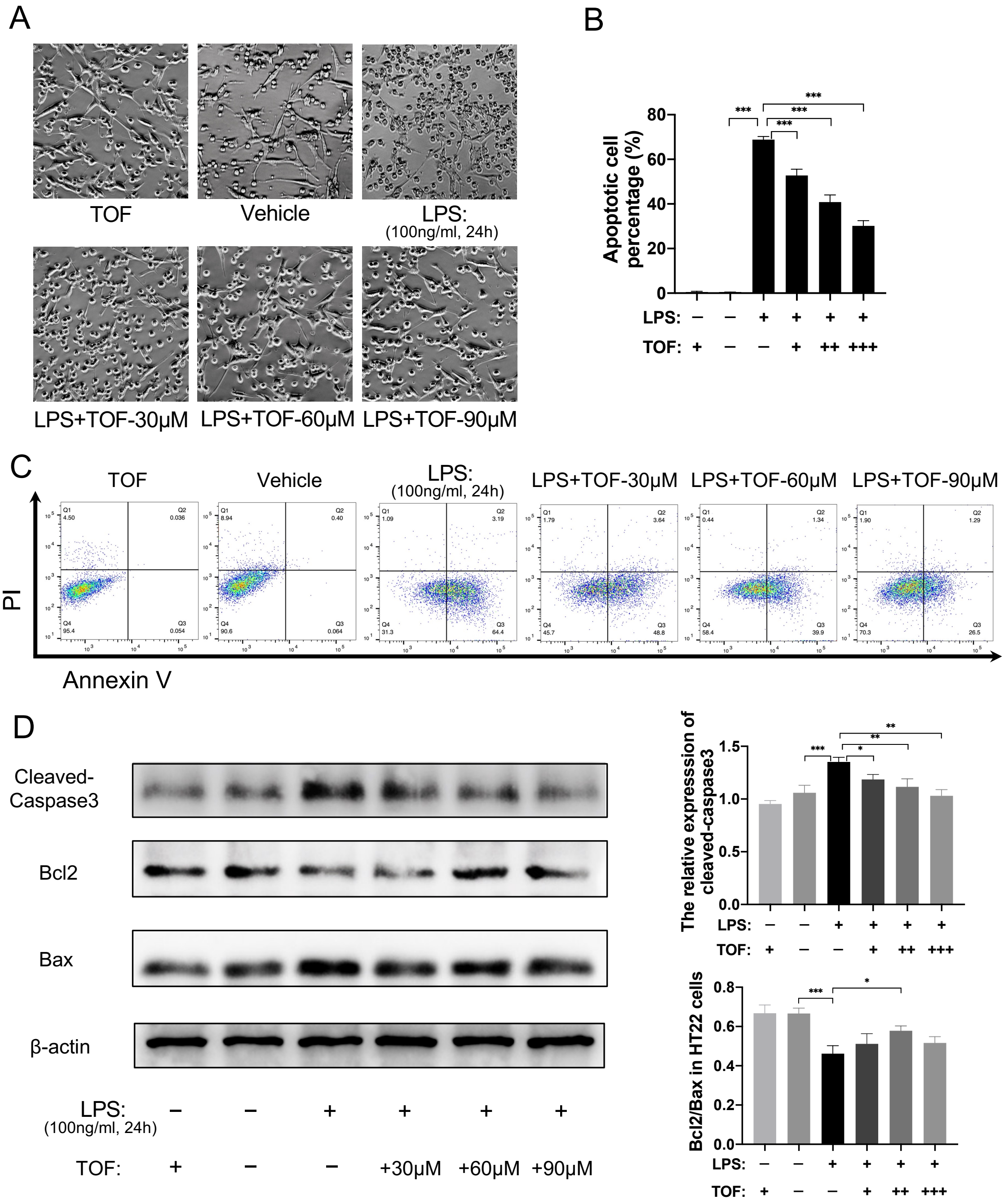

Figure S4. (A) The morphology of HT22 cells after co-culture with BV2 cells. **(B-C)** Flow cytometry analysis of HT22 cell apoptosis in each group (The values are presented as mean ± SD; ***p<0.001, one-way ANOVA). **(D)** Western blot analysis and quantification of caspase-3, Bcl-2 and Bax expression in HT22 cells (The values are presented as mean ± SD; *p<0.05, **p<0.01, ***p<0.001, one-way ANOVA).
